# A sweetpotato gene index established by de novo assembly of pyrosequencing and Sanger sequences and mining for gene-based microsatellite markers

**DOI:** 10.1186/1471-2164-11-604

**Published:** 2010-10-26

**Authors:** Roland Schafleitner, Luz R Tincopa, Omar Palomino, Genoveva Rossel, Ronald F Robles, Rocio Alagon, Carlos Rivera, Cynthia Quispe, Luis Rojas, Jaime A Pacheco, Julio Solis, Diogenes Cerna, Ji Young Kim, Jack Hou, Reinhard Simon

**Affiliations:** 1 Germplasm Enhancement and Crop Improvement Division, International Potato Center, La Molina, Lima, Peru; 2 Research Informatics Unit, International Potato Center, La Molina, Lima, Peru; 3 Genetic Resources Conservation and Characterization Division, International Potato Center, La Molina, Lima, Peru; 4 Department of Horticulture, Louisiana State University Agricultural Center, Baton Rouge, LA 70803, USA

## Abstract

**Background:**

Sweetpotato (*Ipomoea batatas *(L.) Lam.), a hexaploid outcrossing crop, is an important staple and food security crop in developing countries in Africa and Asia. The availability of genomic resources for sweetpotato is in striking contrast to its importance for human nutrition. Previously existing sequence data were restricted to around 22,000 expressed sequence tag (EST) sequences and ~ 1,500 GenBank sequences. We have used 454 pyrosequencing to augment the available gene sequence information to enhance functional genomics and marker design for this plant species.

**Results:**

Two quarter 454 pyrosequencing runs used two normalized cDNA collections from stems and leaves from drought-stressed sweetpotato clone *Tanzania *and yielded 524,209 reads, which were assembled together with 22,094 publically available expressed sequence tags into 31,685 sets of overlapping DNA segments and 34,733 unassembled sequences. Blastx comparisons with the UniRef100 database allowed annotation of 23,957 contigs and 15,342 singletons resulting in 24,657 putatively unique genes. Further, 27,119 sequences had no match to protein sequences of UniRef100database. On the basis of this gene index, we have identified 1,661 gene-based microsatellite sequences, of which 223 were selected for testing and 195 were successfully amplified in a test panel of 6 hexaploid (*I. batatas*) and 2 diploid (*I. trifida*) accessions.

**Conclusions:**

The sweetpotato gene index is a useful source for functionally annotated sweetpotato gene sequences that contains three times more gene sequence information for sweetpotato than previous EST assemblies. A searchable version of the gene index, including a blastn function, is available at http://www.cipotato.org/sweetpotato_gene_index.

## Background

Sweetpotato (*Ipomoea batatas *(L.) Lam.) belongs to the *Convolvulaceae *(morning glory family). With an annual global production of 110 million tons, it is an important crop in developing countries and has an abundance of uses, ranging from consumption of fresh roots or leaves to processing into animal feed, starch, flour, candy, and alcohol. Sweetpotato produces more food per hectare than wheat, rice, or cassava, which makes it an important food security crop. The high pro-vitamin A content of orange-fleshed sweetpotato varieties plays a crucial role in mitigating vitamin A deficiency that is still widespread among the poor in developing countries. Adding orange-fleshed sweetpotato to the daily diet could prevent vitamin A deficiency-related blindness and maternal mortality [1].

African farmers produce about 14 million tons of sweetpotato annually with yields of about 4.2 t/ha. Yields in Africa amount to about a fifth of those in China, indicating a huge potential for future growth [2]. Main reasons for low yields in Africa are, besides lack of high-quality planting material, fertilizers and irrigation, prevailing insect pests, and viral diseases [3]. Genomic tools such as molecular markers could support breeding efforts to produce varieties with high nutritional value and improved virus and insect resistance that would ensure food security of resource-poor farmers in developing countries.

Sweetpotato breeding is constrained by the complexity of the genetics of this out-crossing hexaploid crop and by the lack of genomic resources. Up to now, sweetpotato sequence information has been limited to some 22,000 expressed sequence tag (EST) sequences and ~ 1,500 GenBank sequences and one bacterial artificial chromosome sequence deposited in public databases. At the Plant Gene Database http://www.plantgdb.org/download/download.php?dir=/Sequence/ESTcontig/Ipomoea_batatas/current_version and at TIGR Plant Transcript Assemblies http://plantta.jcvi.org/index.shtml, assemblies of GenBank-deposited *Ipomoea batatas *ESTs are available. More assembled EST sequences are available from morning glory (*Ipomoea nil*), a wild relative of cultivated sweetpotato http://compbio.dfci.harvard.edu/tgi/cgi-bin/tgi/gimain.pl?gudb=morning_glory that might be of use as a model plant for some aspects of sweetpotato genomics. Sweetpotato genomics tools are currently restricted to a medium-density cDNA microarray http://www.picme.at/index.php/products/arrays/?task=details&species=Sweetpotato%20Microarray%20PICME_SWP_15K_1, linkage maps [4,5], and some 130 published microsatellite (SSR) markers [6-10]. To prepare the path for using the large biodiversity of this crop in molecular breeding of improved cultivars, more genomic resources such as sweetpotato gene sequences and markers are urgently needed.

CIP holds in trust 7,783 sweetpotato accessions, including breeding lines, improved varieties, landraces, and wild accessions from 58 countries. A subset of this collection, consisting of 472 accessions that represent the diversity of this crop with respect to agronomical and resistance traits as well as nutritional quality--referred to as a "composite genotype set"--is available for international distribution in the form of disease-free in vitro plants to facilitate access of breeders to sweetpotato biodiversity http://gcpcr.grinfo.net/files/cr_files/gcpcr_file832.xls. The sweetpotato clone *Tanzania*, a member of the composite genotype set, is a high-yielding, stress-tolerant, and broadly cultivated African landrace that is used in various breeding programs due to its high dry matter content and drought tolerance. This clone has been submitted to transcriptome sequencing in the present work, with the aim to augment the available gene sequence and marker information for this crop.

454 pyrosequencing has become a popular method for high throughput sequencing. At low cost, it provides a huge amount of sequence information at relative low error frequency [11]. It is widely used for genome resequencing [12], de novo sequencing of small genomes [e.g., [13]], SNP detection [14,15], or transcriptome sequencing [16]. Specialized software for de novo assembly of the relative short 454 sequences, ranging from around 100 bp with the 454 FLX system but now approaching 500 bp with the 454 FLX TITANIUM system, is available. However, only a few software packages claim to be suitable for hybrid assembly of the relatively short 454 reads with Sanger sequences [17-20]. The choice of optimal assembly parameters to significantly reduce redundancy of an EST collection and, at the same time, maintain paralogous sequences separated in different contigs remains a challenging task. Particularly in a highly heterozygote hexaploid plant such as sweetpotato, discrimination between paralogs and allelic variants on whole transcriptome level remains difficult. For an EST assembly, it might therefore be preferable to accept an increased level of redundancy instead of risking the merging of different members of a gene family into one contig by using too loose assembly parameters. The quality of an assembly generally augments with increasing sequence information. Therefore assemblies with a limited number of ESTs represent a work in progress rather than a finished transcriptome assembly. As more sequence information becomes available, reassembly can lead to splitting or rearrangement of contigs, thus leading to an optimized representation of the transcriptome.

Here we present the de novo assembly of 454 sequencing reads from two normalized sweetpotato libraries, together with publically available ESTs, which resulted in a gene index for hexaploid sweetpotato. This gene index facilitates the access to sweetpotato gene sequences and has allowed for the design of gene-based microsatellite markers.

## Results and Discussion

### 454 sequencing

A total of 87,307 raw reads comprising 21,292,096 bases were obtained with a 454 FLX quarter run of a normalized cDNA library of leaves from drought-stressed plants of the sweetpotato clone *Tanzania*. Another quarter run of 454 FLX TITANIUM resulted in 436,817 raw reads and 136,844,411 bases for a normalized cDNA library of stems of the same clone. The average length per read amounted to 243.9 bp for the 454 FLX run and to 313.3 for the 454 FLX TITANIUM run. cDNA synthesis primer sequences present in the reads were removed and very short reads with less than 100 bp and low-quality sequences were eliminated. The remaining 402,506 raw reads were blasted against vector-cleaned sweetpotato ESTs from the GenBank. Blast hits at > 80% coverage and > 80% identity were found in 31.6% of the 454 sequences, suggesting that the remaining two-thirds (68.4%) of the 454 reads represent new EST sequences.

### Sequence assembly

**Figure 1:**
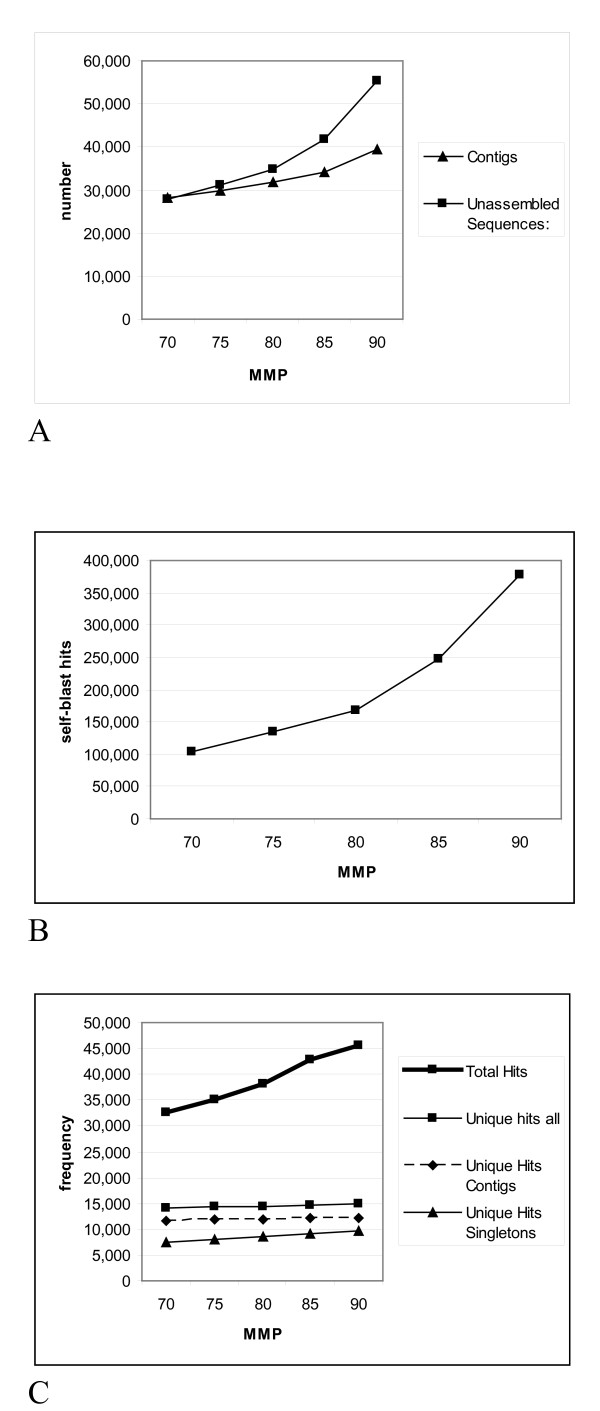
**Variation of the assembly parameter minimum match percent (MMP) from 70 to 90%**. (A) Number of obtained contigs and singletons, (B) number of obtained blastclust clusters, (C) number of blastx-hits with the *A. thaliana *proteome database obtained by varying the assembly parameter minimum match percent (MMP) from 70-90%.

The 402,506 raw reads were assembled together with 22,094 ESTs from the GenBank. We attempted to optimize the assembly parameters by stepwise variation of the minimum match percent (MMP) parameter from 70 to 90% and testing for good representativeness and minimal redundancy of the gene index (Figure 1A-C). The contig number obtained changed linearly and slightly when the MMP parameter was increased from 70 to 80%. From 80 MMP on, the increment of singleton number became steeper and the number of contigs augmented stronger from 85 MMP on (Figure 1A).

Redundancy in the gene index was assessed by self-megablast and blastx with a *A. thaliana *protein database. At the nucleic acid level, we performed a self-megablast test to assess similarity between contig and singleton sequences produced at different MMPs. Sequences sharing 80% identity over 80% of their length with another sequence were considered as redundant. As expected, self-megablast hits augmented with increasing MMPs, indicating that sequences representing allelic variants increasingly remained unassembled or were resolved into different contigs. The increase in the number of self-megablast hits went in parallel with the number of sequences and became steeper at MMPs above 80% (Figure 1B). Additionally, we have assessed the redundancy of the assemblies produced at different MMP levels by counting the number of total and unique blastx hits of the contig and singleton sequences with a protein database. At this stage, in order to make redundancy analysis quicker and less demanding with respect to computer power, we used the *A. thaliana *protein database instead of the very large UniRef100 protein set for blastx analysis. Predictably, the total number of blastx hits increased with rising MMP and the increase became steeper above 80% MMP, whereas unique blastx hits increased linearly over the whole range of MMP and at a much lower path than the total hit number. The number of hits increased mainly for singletons, as more sequences remained unassembled at higher MMP values (Figure 1C).

**Figure 2:**
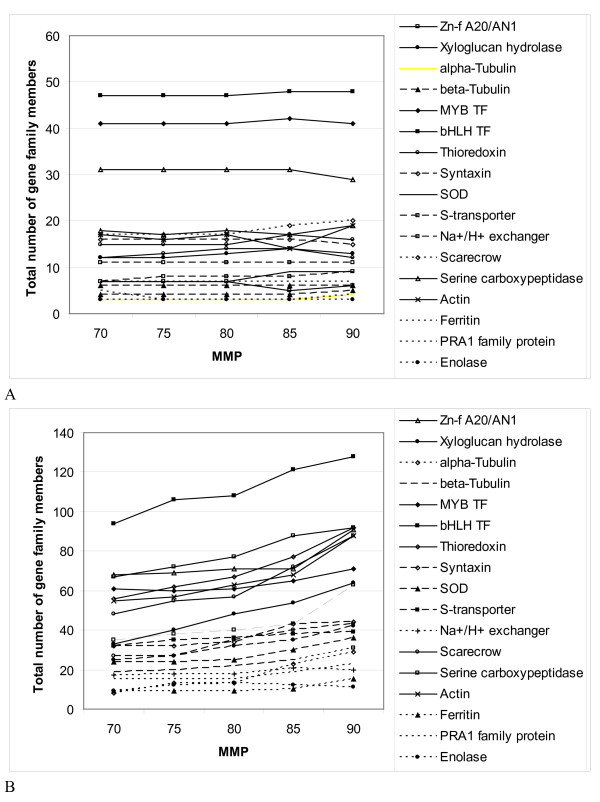
**Assessment gene family member changes at different MMP**. Twenty selected gene families (Zinc finger A20 and AN1 domain-containing stress-associated protein, xyloglucan endotransglucosylase/hydrolase protein, α-tubulin, β-tubulin, MYB, bHLH, thioredoxin, syntaxin, superoxide dismutase, sulfate transporter, Na^+^/H^+ ^exchanger, scarecrow, serine carboxypeptidase, actin, ferritin, PRA1 family protein, enolase, β-amylase, bZIP, and glutaredoxin) were analyzed for the total number of members (A) and the number of different members (B) represented in the gene index at 70-90 MMP. While at higher MMP the total number of gene family members increased strongly due to augmentation of the redundancy in the gene index, the representation of different gene family members in the index (B) hardly changed with increasing MMP.

To investigate whether assembly at low MMP results in merging of reads of paralogous sequences into one contig, we have analyzed the number of total and different members of 20 randomly chosen gene families in the assemblies at different MMP (Figure 2A and 2B). In a simple text search using uniprot identifiers and text annotation, we have looked for sequences annotated as Zinc finger A20 and AN1 domain-containing stress-associated protein, xyloglucan endotransglucosylase/hydrolase protein, α-tubulin, β-tubulin, MYB, bHLH, thioredoxin, syntaxin, superoxide dismutase, sulfate transporter, Na^+^/H^+ ^exchanger, scarecrow, serine carboxypeptidase, actin, ferritin, PRA1 family protein, enolase, β-amylase, bZIP, and glutaredoxin according to sequence comparison with proteins of the *A. thaliana *proteome. Although the total count of members over the 20 gene families increased from 734 at 70 MMP to 1,100 at 90 MMP, the number of different gene family members varied little over the different MMP--from 286 to 296--indicating that the risk of merging paralogs into one contig at low MMP exists but remains low. Therefore, to ensure both good representativeness and low redundancy and, at the same time, to mitigate the risk that reads derived from paralogous sequences are assembled together into one contig, we chose the medium stringent 80 MMP as assembly parameter for the sweetpotato gene index. At 80 MMP the assembly still had relative low redundancy, while the representativeness was better than at lower MMP values, indicated by 400 more unique blastx hits with the *A. thaliana *proteome than at 70 MMP.

**Figure 3:**
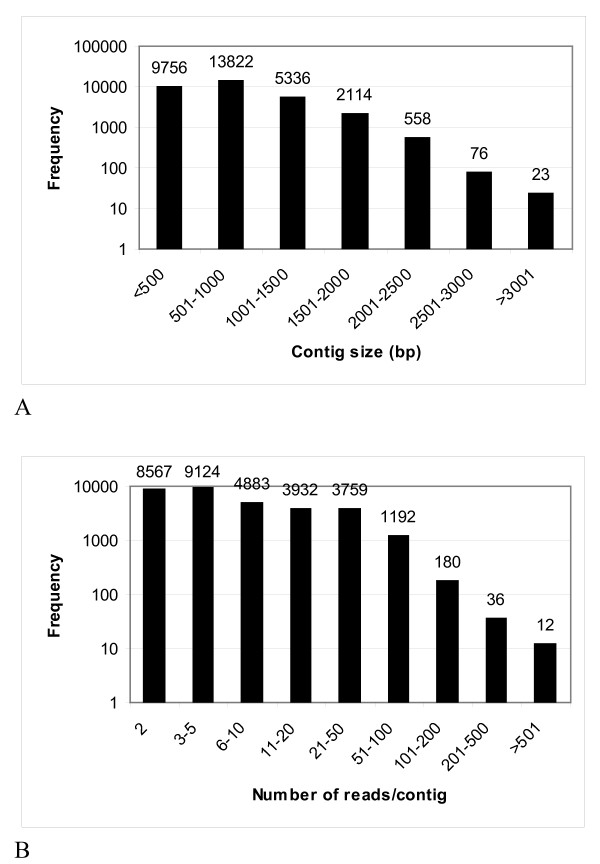
**Contig size and coverage**. (A) Size distribution and (B) number of reads per contig in the final assembly.

Hybrid assembly of our 454 reads with EST sequences obtained from the GenBank at 80 MMP produced 31,685 contigs and 34,733 reads remained as singletons. Contigs ranged from 100 to 6,872 bp in size, with an average of 790 bp. The cumulative length of the contigs was 25,048,392 bp. The size distribution for the contigs is shown in Figure 3A. A substantial number of large contigs was obtained: 21,929 contigs were > 500 bp in length, and 8,107 were > 1 kb. Sequencing coverage ranged from 2 to 1,863 reads per contig, with an average coverage of 12.3 (Figure 3B).

### Reciprocal blastn queries with previous *Ipomoea *EST assemblies

**Figure 4:**
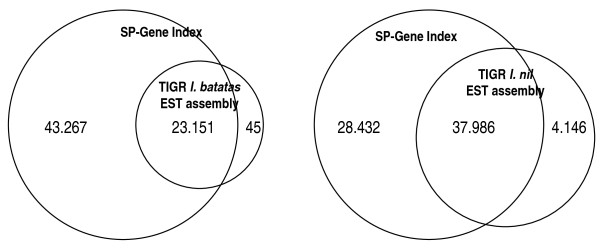
**Coverage of previous *Ipomoea *EST assemblies by the present gene index**. Venn diagrams showing the overlap between the present sweetpotato gene index and a previous *I. batatas *EST assembly (A) and an *I. nil *gene index (B). Most sequences of the previous assemblies are represented in our gene index.

An assembly of GenBank-available EST and other sequences of *I. batatas *consisting of 2,472 contig and 6,695 singletons has been released previously [21]. Sequence comparison to this assembly gives an estimate of the amount of novel sequences in the present gene index. Our expectation was that, in addition to new sequences, our gene index should cover all contigs and singletons of the previous assembly, as the EST sequences used in the previous assembly also were co-assembled with the new 454 reads. And indeed, all but 45 out of 9,167 sequences of the previous assembly had hits in our gene index. Of the 45 no-hit sequences, 22 corresponded to GenBank mRNA sequences that were not included as input for our assembly, and 23 other sequences corresponded to singletons of GenBank-derived ESTs of bad quality or with highly repetitive sequences that were filtered out by our sequence cleaning process. A total of 23,151 sequences of the present gene index had significant similarity to 9,166 contigs and singletons of the plant EST assembly. This indicates that redundancy in our gene index is about 2.5 times higher than in the previous assembly. No significant similarity to the previous EST assembly was found in 43,267 gene index sequences that are considered to represent new sequences (Figure 4). Similarly, only 33 sequences of the PlantGDB sweetpotato EST assembly were not contained in our gene index (not shown). Assuming a redundancy of 2.5-fold of our gene index, the present gene index would contain about three times more sequence information for sweetpotato genes than the previous EST assemblies.

For *I. nil*, a near relative of sweetpotato, a gene index has been established based on 61,199 EST and 133 mature transcripts (ETs), resulting in 11,754 tentative consensus sequences (contigs), 9,721 EST-singletons, and 39 ET singletons [22], http://compbio.dfci.harvard.edu/cgi-bin/tgi/gimain.pl?gudb=morning_glory. The present gene index had 37,986 significant (E = 10^-6^) blastn hits with the *I. nil *database, while 28,432 sequences remained without a hit and can be considered as specific for sweetpotato. About 19% of the sequences of the *I. nil *gene index (4,146 contigs and singletons) had no significant hits with the present sweetpotato gene index. These reciprocal blastn tests indicate good coverage of the present gene index for sweetpotato, but also a relatively high redundancy compared to previous EST assemblies.

### Sequence annotation

**Figure 5:**
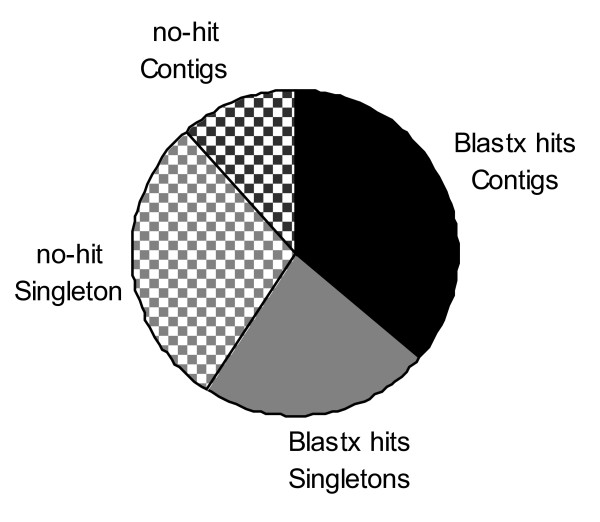
**Distribution of blastx hits and no hits over contigs and singletons**.

Of the 66,418 sequences, 39,299 (59%) of the gene index sequences had significant blastx matches (E = 10^-10^) with sequences of the UniRef100 protein database. These matches corresponded to 24,657 different unique UNIRef100 accession numbers. The frequency of sequences with significant blastx hits was higher in contigs than in singletons (Figure 5). From 7,728 contigs without significant hits to proteins of the UniRef100 database, 2,829 contained a putative poly-A tail and thus most probably represent a part of a transcribed gene. From the remaining 4,899 contigs, 3,464 had an open reading frame (ORF, counted from a possible methionine start codon to a stop codon) encoding at least 50 amino acids. This suggests that most contigs without blastx hits are in fact derived from protein coding genes.

The lower hit frequency of singletons in blastx searches might be caused by the smaller size of these sequences compared to contigs (373 vs. 790 bp). Another possibility for decreased hit frequency of singleton sequences would be contamination of the cDNA preparation with genomic DNA, resulting in reads derived from genomic DNA that remained unassembled. Analogous to the contig sequences, we also have searched for the presence of poly-A tails and ORFs in singletons without significant blastx hits. From 19,391 singletons without significant similarity to any protein of the UniRef100 database, 6,775 had a putative poly-A tail. Only 635 singletons of the no-hit singletons, including 47 ESTs from the GenBank database, did not encode any ORF larger than 10 amino acids, while the remaining no-hit singletons had ORFs from 30 to 896 bp. The presence of ORFs in more than 90% of the un-annotated singletons suggests that these sequences also are derived from protein coding genes.

The sweetpotato gene index including the annotation of the contigs and singletons can be viewed, searched, and downloaded at http://www.cipotato.org/sweetpotato_gene_index.

Not all contigs and singletons, however, were derived from sweetpotato: several sequences were clearly identified as viral sequences (Tab. 1). These viral sequences were derived from sweetpotato feathery mottle virus, sweetpotato chlorotic stunt virus, sweetpotato leaf curl virus, or viruses with similarity to potato virus A and petunia vain clearing virus that apparently have been present in the experimental plants. Also, two singleton sequences derived from Sanger ESTs downloaded from the GenBank represented viral transcripts (Tab. 1).

**Table 1:** Viral sequences contained in the sweetpotato gene index

Contig/singleton ID	length	ID	E-value	Text annotation
03767	1851	Q59A02	0.0	Polyprotein (Fragment) n = 1 Tax = Sweet potato feathery mottle virus RepID = Q59A02_9POTV
10592	1329	Q6XKE6	6e-49	Reverse transcriptase n = 1 Tax = Petunia vein clearing virus isolate Hohn RepID = POLG_PVCV2
12069	526	O39734	3e-90	Polyprotein n = 1 Tax = Sweet potato feathery mottle virus RepID = O39734_9POTV
12293	504	UPI0000163D32	6e-67	P3 protein n = 1 Tax = Sweet potato feathery mottle virus RepID = UPI0000163D32
13375	686	O39734	2e-84	Polyprotein n = 1 Tax = Sweet potato feathery mottle virus RepID = O39734_9POTV
23606	528	Q9QS58	1e-101	Coat protein AV1 n = 1 Tax = Sweet potato leaf curl virus RepID = Q9QS58_9GEMI
27246	614	Q9QBT4	2e-67	Polyprotein n = 1 Tax = Potato virus A RepID = Q9QBT4_PVMA
27646	725	UPI0000156FE9	1e-140	NIb protein n = 1 Tax = Sweet potato feathery mottle virus RepID = UPI0000156FE9
29621	347	Q9QS57	2e-47	AC3 n = 1 Tax = Sweet potato leaf curl virus RepID = Q9QS57_9GEMI
131893_1741_0589-I	221	UPI0000163D38	9e-36	coat protein n = 1 Tax = Sweet potato feathery mottle virus RepID = UPI0000163D38
158451_1257_3402-I	250	B3Y549	4e-37	AV2 protein n = 1 Tax = Sweet potato leaf curl virus RepID = B3Y549_9GEMI
DC882409.1	418	Q80MW4	2e-28	Polyprotein (Fragment) n = 1 Tax = Sweet potato feathery mottle virus RepID = Q80MW4_9POTV
EE874850.1	669	Q8JJW9	1e-106	Polyprotein n = 1 Tax = Sweet potato chlorotic stunt virus RepID = Q8JJW9_9CLOS
FRFM4LP02P1T45-II	318	UPI0000163D31	5e-34	HC-Pro protein n = 1 Tax = Sweet potato feathery mottle virus RepID = UPI0000163D31
FRFM4LP02QRY4N-II	502	Q5GIU0	5e-45	Polyprotein (Fragment) n = 1 Tax = Sweet potato feathery mottle virus RepID = Q5GIU0_9POTV
FRFM4LP02R1W0W-II	382	UPI0000163D34	4e-64	CI protein n = 1 Tax = Sweet potato feathery mottle virus RepID = UPI0000163D34
FRFM4LP02RT0LA-II	328	C4N332	2e-36	Polyprotein n = 1 Tax = Sweet potato feathery mottle virus RepID = C4N332_9POTV
FRFM4LP02RUC7U-II	451	O39734	4e-73	Polyprotein n = 1 Tax = Sweet potato feathery mottle virus RepID = O39734_9POTV

**Figure 6:**
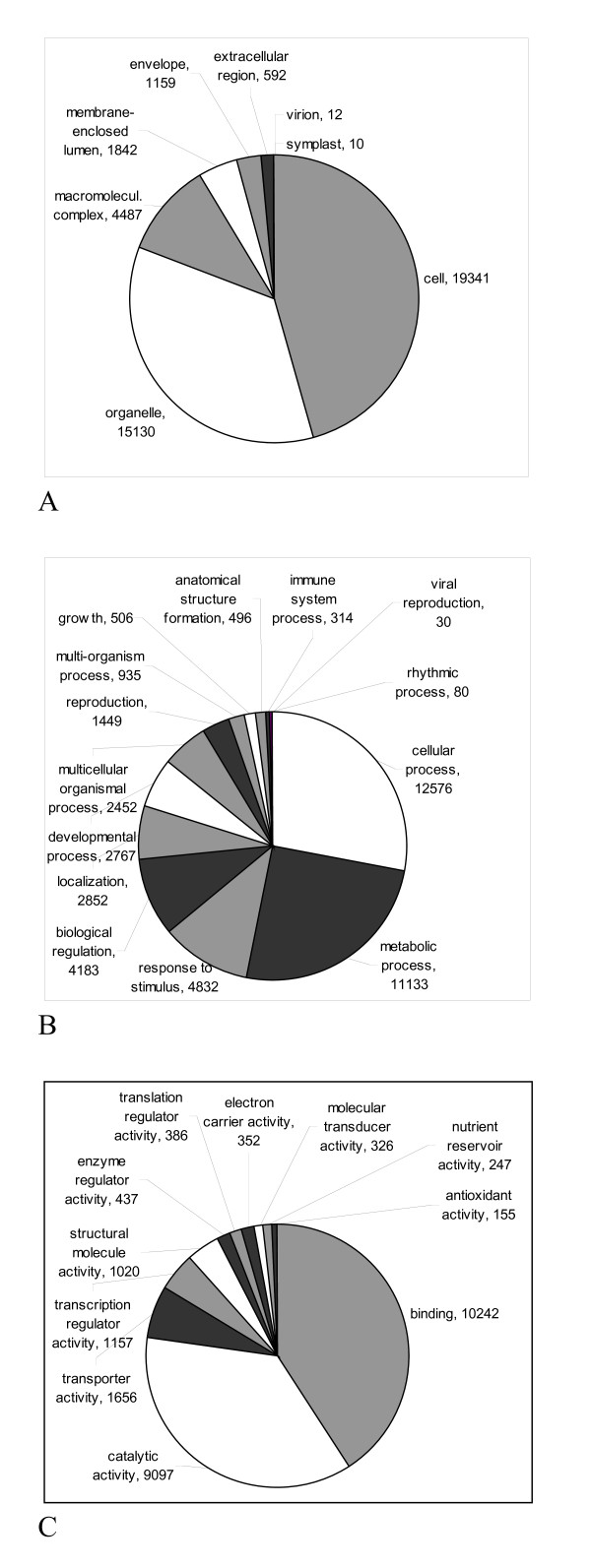
**GO-annotation**. (A) Cellular location, (B) biological function, (C) molecular function.

A total of 24,763 out of the 39,299 blastx-annotated sequences gene ontology (GO) terms could be associated. We annotated the gene index sequences at level 2 for the three main GO vocabularies: cellular component, biological process, and molecular function (Figure 6A-C).

### Microsatellite marker identification and testing

Microsatellites for sweetpotato have been identified in ESTs previously [10]. To ensure identification of new microsatellite motifs only, sequences similar to GenBank ESTs were excluded from the search. A total of 1,621 new SSR motifs have been identified in the gene index, including 936 di-, 581 tri-, and 124 tetra-nucleotide SSR motifs with a minimum of 7, 5, and 5 contiguous repeating units, respectively. For SSR testing, we have taken into account the sequence redundancy of the gene index and selected only those SSRs that were present either in a single contig or singleton or in a group of homologous sequences that were derived most probably from a single locus. This analysis resulted in 223 SSR loci, for which high-quality primers were designed. Amplification of the 223 SSR loci was tested in eight sweetpotato clones, consisting of six hexaploid and two diploid accessions (Tab. 2). From the 223 selected SSR loci, 195 yielded amplification products in the predicted size, 150 SSRs revealed to be polymorphic in the eight-clone test set, and 26 were monomorphic representing in total 729 alleles (mean 4.1 alleles/locus, Additional file 1). None of the SSRs showed more than 2 alleles in any of the diploid test accessions but up to six alleles in hexaploid sweetpotato. For 19 SSR primer pairs that successfully amplified fragments from genomic DNA, polymorphism tests have not been performed.

**Table 2:** Sweetpotato accessions used for SSR testing

CIP number	Species	Cultivar Name	Origin	Description
107665.9	*Ipomoea trifida*	M9	CIP	diploid mapping parent
107665.19	*Ipomoea trifida*	M19	CIP	diploid mapping parent
401206	*Ipomoea batatas*	401206	Mexico	hexaploid, landrace
420027	*Ipomoea batatas*	Zapallo	Peru	hexaploid, landrace
440025	*Ipomoea batatas*	Xushu 18	China	hexaploid, improved variety
440131	*Ipomoea batatas*	Naveto	Papua New Guinea	hexaploid, landrace
440132	*Ipomoea batatas*	Beauregard	USA	hexaploid mapping parent, improved variety
440166	*Ipomoea batatas*	Tanzania	Uganda	hexaploid mapping parent, landrace

## Conclusions

The present gene index http://www.cipotato.org/sweetpotato_gene_index strongly augments the available sequence information on sweetpotato genes. Owing to the heterozygous and hexaploid nature of this plant, the assembly contains a relatively high level of redundancy compared to previous assemblies to prevent merging of paralogous sequences into contigs. Fifty-nine percent of the obtained sequences could be functionally annotated. The index has been successfully mined for microsatellite sequences and 195 out of 223 selected loci could be successfully amplified.

## Methods

### Plant material, cDNA synthesis, 454 sequencing

Shoots from field-grown plants of the sweetpotato *I. batatas *variety *Tanzania *(CIP accession number 440160) were acclimated to the greenhouse and cultivated in pots for one month. Drought was then imposed by not watering the plants for eight weeks. After that time, leaf and stem tissue were sampled separately and total RNA was produced using the Trizol reagent according to the instructions of the supplier (Invitrogen). Complementary DNA was synthesized from the two RNA batches by Evrogen http://www.evrogen.com using the SMART approach [23], normalized according to [24], and amplified. Seven μg DNA of each normalized cDNA library was submitted to a quarter 454 sequencing run at the School of Biological Sciences, University of Liverpool. The cDNA library of leaves was sequenced with the 454 FLX technology and the stem library was sequenced using 454 FLX TITANIUM.

### Sequence cleaning and assembly

Sequence cleaning and assembly were performed on the CIP High Performance Computer http://hpc.cip.cgiar.org/. Adaptor primer and SMART oligonucleotide sequences as well as low-complexity regions present in the 454 raw reads were masked using the open source software RepeatMasker 3.2.7 http://www.repeatmasker.org/RMDownload.html. Short reads (< 100 bp) were eliminated from the sequence and quality files using custom scripts. A total of 20,094 publically available sweetpotato EST sequences http://www.ncbi.nlm.nih.gov/Genbank/ were downloaded from http://www.ncbi.nlm.nih.gov/sites/entrez and cleaned from vector sequences using SeqClean http://compbio.dfci.harvard.edu/tgi/cgi-bin/tgi/download.pl?ftp_dir=software&file_dir=seqclean/seqclean.tar.gz. The 454 reads were assembled together with the GenBank ESTs with the NGen software (DNASTAR, Madison, WI, USA). For optimization of the assembly, the assembly was tried at MMP in a range from 70 to 90% with 5% intervals. The final assembly was done using the following parameters: match size: 25, gap penalty: 7, mismatch penalty: 12, match score: 10, minimal match percentage: 80, and match spacing: 40. The quality of the assembly was assessed manually for 300 randomly selected contigs by checking the plausibility of the read alignments. Redundancy of the assembly was checked by selfblast using megablast (NCBI) on the CIP High Performance Computer http://hpc.cip.cgiar.org.

### Sequence annotation

Contigs and singletons were annotated through blastx sequence comparison to the UniRef100 database http://www.uniprot.org and *A. thaliana *proteins (TAIR, http://www.arabidopsis.org) using BLAST 2.2.14 on the CIP High Performance Computer http://hpc.cip.cgiar.org. The best BLAST hits were filtered out with the custom R-script Chomper2.R. GO annotation was performed using Blast2GO software v2.4.2 [25,26] with a blastx cutoff of 10^-6^.

### Identification of open reading frames

Open reading frames in contig and singleton sequences without significant blastx hit were identified using Orf-Predictor http://proteomics.ysu.edu/tools/OrfPredictor.html.

### Microsatellite marker identification and testing

Gene index sequences that had high similarity to GenBank-deposited EST sequences were filtered out to avoid identification of already known SSR sequences. SSR motifs were identified with the SSRlocator [27], http://minerva.ufpel.edu.br/~lmaia.faem/ssr1.html limiting the hits to motifs that consisted of at least 7 dimers, 5 trimers, or tetramers. Primers for SSR loci were designed with Primer3 with 100-200 bp amplicon size. The primer sequences of the identified SSR loci were compared via blastn with the gene index sequences. Only those primers that gave an unambiguous hit with one contig or singleton or "single locus sequence group" were recorded as candidate SSR. A single locus sequence group was defined as a group of sequences that, based on sequence similarity, was putatively derived from a single locus. We assumed sequences to represent a single locus if the distal sequences were identical. SSR loci were polymerase chain reaction (PCR) amplified in six biodiverse hexaploid sweetpotato and two diploid wild sweetpotato accessions. Four of these accessions represented clones from Asia, Latin America, and Africa, and four accessions were parents of mapping populations (Tab. 2). DNA extraction was done according to [28] for SSR analysis, and PCR reactions contained 1x PCR-buffer with 2 mM MgCl_2_, 0.6 mM dNTPs (New England Biolabs), 0.5 μM forward and reverse primer, and 0.5 U Taq polymerase (New England Biolabs) in a volume of 25 μl. Amplification conditions were 4 min. initial denaturation followed by 30 cycles of 1 min at 94°C, 1 min. at annealing temperature (given with the description of the SSR primers, 'Additional file 1), 1 min. at 72°C, and followed by terminal elongation at 72°C for 7 min. The obtained fragments were separated on a Li-Cor 4300 (LI-COR Biosciences, Lincoln, NE, USA) and analyzed essentially as described in [29].

## Authors' contributions

LRT produced the RNA for cDNA synthesis, performed the 454 sequence cleaning, and performed together with RoS the sequence assembly and annotation and participated together with GR, CR, RR, JYK, CQ, DC, RA, and JS with the SSR marker design and testing. ReS, OP, and JPa produced the format of the gene index database and provided bioinformatics expertise. LR, JS, and JH produced perl and R scripts for sequence analysis and annotation. The manuscript was produced by RoS and was approved by all authors.

## Supplementary Material

Additional file 1 **Sweetpotato SSR marker: Primer sequences and SSR motifs identified in the sweetpotato gene index and successfully amplified**. Click here for file
