# Supplementary material for: A sweetpotato gene index established by de novo assembly of pyrosequencing and Sanger sequences and mining for gene-based microsatellite markers
Source: BMC Genomics. 2010 Oct 26;11:604. doi: 10.1186/1471-2164-11-604 (PMC3017860; doi:10.1186/1471-2164-11-604)
Supplement: Additional file 1 — Sweetpotato SSR marker: Primer sequences and SSR motifs identified in the sweetpotato gene index and successfully amplified. [file 1471-2164-11-604-S1.DOC]

## Additional file 1 – Sweetpotato SSR marker: Primer sequences and SSR motives identified in the sweetpotato gene index and successfully amplified

| Primer Name | Forward primer | Reverse Primer | SSR motive | Annealing temp. | Product length | # alleles | polymorph (p) monomorph (m) | Contig/singleton ID |
| --- | --- | --- | --- | --- | --- | --- | --- | --- |
| IbL1 | TTTAGTCCCAGTATGACAATGC | GAAAGAACAGAGATCCTTCGAC | (AGA)6 | 60 | 156 | 2 | p | 17046 |
| IbL10 | TCGACGAGTGATCTGTAATCAT | GGAGAATCACACTCCATCATC | (CTC)6 | 60 | 151 | 2 | p | 21539 |
| IbL11 | GGGAGGGTTATAATTGTGGAC | GAAACAATTTCCTCCACCAC | (GCG)6 | 60 | 185 | 3 | p | 28809 |
| IbL12 | CGATGGACAGAACAACTACTTC | AAATAGCCTAAGACCAGAACCA | (AT)6 | 60 | 110 | 2 | m | 21624 |
| IbL13 | ACGAGAAGACCGAGAAACAG | CCTATACGATAAGAATGGCTGG | (GCG)6 | 60 | 125 | 2 | m | 06642 |
| IbL14 | TCTTCATCTCATCATCATCCTC | CTTCCTCGTACCTGCTAAGAAT | (TTG)6 | 60 | 103 | 3 | p | 09103 |
| IbL15 | AACCACCTCAGACTCCTAAAAA | TTTATCCACTCTCACAATCTGC | (AGG)6 | 60 | 152 | 3 | m | 17623 |
| IbL16 | GTCTTGCTGGATACGTAGAACA | GGGAGAAGTAAGAGAACCGATA | (TTC)7 | 60 | 161 | 9 | p | 21913 |
| IbL18 | GATTCAAATGGGGAGAATTG | GAGCTTCAATATCATTTTCTCTGA | (ATT)6 | 60 | 175 | 3 | p | 24109 |
| IbL2 | TCGTGCATCATGTTTAGGTC | CCAACATGAATATGTTCAAACG | (GAA)6 | 60 | 177 | 5 | p | 09150 |
| IbL20 | CTTCTACGACCAACTCTGATGA | AACTTTGTAGAGAAGATTCGGG | (AAG)6 | 60 | 156 | 3 | p | 23608 |
| IbL21 | GTTTTCTCGACGAAACCATT | TAACCAGAAGAGGAAACCCTAA | (GCC)6 | 60 | 127 | n.d. | n.d. | 23092 |
| IbL22 | GTCCGTCGATAAACACTACAAG | GTTCTGTGAGATGTGAATTTCC | (TCT)6 | 60 | 150 | 2 | p | 20973 |
| IbL23 | CCAGTCAGTCACATCTCTCAAG | GCCAGAATCGAAGACTATATCC | (GAT)6 | 60 | 177 | 5 | p | 13700 |
| IbL24 | AAAAATGTACTCGGCGGTGC | GGTGAGAACAAGACAAGCATC | (ACC)6 | 60 | 142 | 2 | p | 28849 |
| IBL25 | TTCTTCACTCCACGTCAGTAAC | TGGGTAGAGAAATGTGTCTTTG | (CTT)8 | 60 | 168 | 4 | p | 24401 |
| IbL31 | GTTCAAAACAGTTGGGTGAGA | AGGAACGCACAGAGACTTTATT | (ATC)6 | 60 | 159 | 2 | p | 16720 |
| IbL32 | GGGATGAAGGAGAGAATGAGTA | TTGAAAACCTAGAGAGAAAGGG | (TGA)8 | 60 | 158 | 9 | p | 1510/13512 |
| IbL33 | TCTTGTCGAGTACTTCAGATGG | TCTATGTCGGTCTGATGATGTT | (CAA)6 | 60 | 174 | 4 | p | 04750 |
| IbL34 | ATAAATCATATCCCATGGAGGG | TCATATGATCTCTATTGCGGAG | (TTA)6 | 60 | 169 | 3 | p | 003150_1297_3371 |
| IbL35 | GAGTTCTACATCGACACCGAC | GAACTCTACAGCTCATGACGAA | (TCC)6 | 60 | 145 | 5 | p | 011270_1347_2155 |
| IbL36 | CTTCATCTTGGGCTTCCGAT | CTAAGACCTCCAAGATCCGAC | (TTG)8 | 60 | 158 | 3 | p | 017734_1289_0657 |
| IbL37 | TCACACACTTCATATGCCTACA | GGTTCCCGATCTGTGATAATAA | (TCC)7 | 60 | 150 | 2 | p | 029308_1564_1963 |
| IbL40 | CTAATCAGGGAGGACTTCCTTT | GTTACTCATCATTGGGTGGTC | (TGC)6 | 60 | 158 | 4 | p | 043456_1128_3657 |
| IBL41 | CAGATAAAATATCGTCGTGGTC | TATTCCTATCCCTCGTTGATCT | (GAA)7 | 60 | 156 | 3 | p | 04047 |
| IbL42 | AAGCATCGAGTGCGTATCGT | TTAGCGACGGAGTTGTACTGT | (ACC)6 | 60 | 117 | 5 | p | 046515_1487_0122 |
| IbL43 | GATATCTTTTCCACCTCCAAGA | GTGATGAAGCTTTCAGAAGTGA | (GAA)7 | 60 | 138 | 2 | p | 21851 |
| IbL44 | GCTAGGAGTTGGGAAGTCAG | CCAACAGTCATGGCTTCTAATA | (GAA)6 | 60 | 155 | 4 | p | 29325 |
| IbL46 | CTGAAATTAGGGATTGAAGAGG | TCCAATCACTCCTTGTTTTCTC | (AGA)9 | 60 | 171 | 6 | p | 12029/110909_1786_0720 |
| IbL49 | GGCATTTGTAGTACTCCATCCT | AAGAAGAAGAGAACCGCCAT | (TCG)6-(GCC)5 | 60 | 163 | 2 | m | 165955_1573_2579 |
| IbL6 | AGAAGCTGGTGGCTCGTAAC | GATGTACTTTGGAATGCTGATG | (GTC)5 | 60 | 170 | 4 | p | 08403 |
| IbL9 | TGTACAGAATAACCATCCCACT | CAGCCATGACTGTGATAAACTT | (AGA)7 | 60 | 168 | 5 | p | 02234 |
| IbO2 | TGTGGATCTGTTCTTTGAACC | TTCCATGTGGAGTGTGAAGTAT | (CTAT)8 | 60 | 175 | 9 | p | 18128 |
| IbO3 | GATCCTCCAATCTGCACTCTA | TGAATGCGACACTAAAACACT | (GACA)5 | 60 | 157 | 4 | p | 4964/FRFM4LP02RDYSH |
| IbO4 | AATTAGACAGTCGTTTTAGGGC | CGCAGAGTAGATCAAGCATAAG | (ATAC)5 | 60 | 112 | 3 | p | 24990 |
| IbO5 | CGCAAATGACTGATTCTGTACT | CCATTACACTTACAGCTGCTTG | (ACAT)5 | 60 | 161 | 2 | p | 12090/023644_1491_0354 |
| IbO7 | TGAGTGGAGGAACAATGTAAAG | CCAGCACAACAAATTGCTACTA | (AGCC)5 | 60 | 162 | 3 | p | 05712 |
| IBS1 | GATATTCTGTTTGCTGCTCTCA | CGTCATCATCATAATCTTCCTC | (TCC)7 | 60 | 134 | 5 | p | 03529 |
| IBS10 | ATCCATCTCATCCACAACAA | CAACTTAACGGACATACGTACTCTT | (TGCA)5-(ATCC)5 | 60 | 200 | 4 | p | 19622 |
| IBS100 | TGCTATAGTTACGTGGACGAAG | TTTAATGCTGATGTGGATGC | (AAT)7-(AAC)7 | 60 | 167 | 6 | p | FRFM4LP02RC6AP |
| IBS103 | CATTATCTCCATCAACTCCTCC | AGCCTGAGAAGAATGGTAATGT | (CT)10 | 60 | 120 | 4 | p | FRFM4LP02S25FZ |
| IBS104 | CCTATTGGTGTCCTTTGGATT | AAGAAGAAGAGGAAGAAGACGA | (ATC)6-(TCT)5 | 60 | 129 | 3 | p | 12241 |
| IBS106 | ACCAGAAAAGGAAAAGTAGCAG | TGCAAGAAGATTAGTGGACAAC | (AT)11 | 60 | 176 | n.d. | n.d. | FRFM4LP02PNQ89 |
| IBS107 | GCAAGCTGTGGCATTAAATC | GAACCCTAAAAAGAAGAACCCT | (TTC)7 | 60 | 194 | 5 | p | FRFM4LP02T1EVY |
| IBS108 | CTCTTTATTCAAGTTTCCCCAC | CAGGTTGACATGTTTACTGAGC | (TCT)7 | 60 | 184 | 5 | p | FRFM4LP02QV7ZQ |
| IBS109 | GTCGCTATGTGTTGACTGTTG | TTATCCTCAACGTGCAAATG | (AATA)5 | 60 | 136 | 7 | p | FRFM4LP02QEV9O |
| IBS11 | ACCTTCATCCCATCTCTCTCTA | GGAGCTGGAGAAGCTTTAGAT | (TATC)11 | 60 | 128 | 7 | p | FRFM4LP02SNK8Z |
| IBS110 | AGACGGGAAGAATATCAATGAG | TTAGATCCATGTCCTGAGTTTG | (AAG)7 | 60 | 160 | 4 | p | FRFM4LP02RKLTA |
| IBS111 | TGGGATTGTGAACTTGGATAG | ATGATCCATCATCCATCACA | (TGA)9 | 60 | 104 | 2 | m | FRFM4LP02SAMP2 |
| IBS113 | AGCACTAGACTACCTGAGGAGG | TAGCATGCTGCGTCTACAAT | (ATT)7 | 60 | 196 | 3 | p | FRFM4LP02PT9US |
| IBS115 | TATCATTCTACCATGTTGACGC | GATTCCTGGGAAATAATGAAGC | (TTA)8 | 60 | 163 | 2 | p | 02875 |
| IBS117a | GATGGAGGAAGATATTTGGGT | CTTTCTTTCTTTCTGCTTCTGC | (CT)10 | 60 | 110 | 9 | p | 7126/FRFM4LP02SL80T |
| IBS118 | GGTAGAAGACGATGACGAAGAC | AAAATTAGTCCAACTCCCACAC | (ACT)6 | 60 | 197 | 5 | p | 08316 |
| IBS12 | CAGTTATCAATTCCCACCTACC | TTGCTGTGTTATAGGCTTTGTC | (GA)10 | 60 | 170 | 8 | p | 28524 |
| IBS120 | GGAGCTGAAGCTCTCTTACAAT | TGAGGTAGATTGAGCTCTGAAA | (GAT)8 | 60 | 174 | 1 | m | 05224 |
| IBS121 | CTCTTGGTTCTGTGGACTGTAG | GTTAGGAAAGCAGAAGATAGGC | (CAC)8 | 60 | 178 | 2 | m | 02186 |
| IBS122 | CtAGCCATTGTGAATGTGTCTT | CTCGCTCAGATTTCTCTCTTTT | (TTTA)5 | 60 | 198 | n.d. | n.d. | 05391 |
| IBS123 | GGTGGGTATAGGAAGTCATCAT | CACAACTTGATCCTGTATCGTC | (ATG)8 | 60 | 112 | 1 | m | 06415 |
| IBS126 | TACACATTCGCCTACAGATTTC | GGGAAGACGTAGTAGGTCGTAG | (TC)14 | 60 | 126 | 2 | p | 18181 |
| IBS129 | ATATGTACCCACACCCCTATGT | GATACCAGGTGAGTAGGTGGTT | (AG)10 | 60 | 125 | 8 | p | 07188 |
| IBS13 | ATCCATGGTGGGGTACTAGACT | CCTATGTTTGTGAGAATGGACA | (TAT)5 | 60 | 195 | 9 | p | 16873 |
| IBS132 | GATCGAGAGCATCACAGATAAA | TTCACGTAAAGATATGGAACCC | (AAC)7 | 60 | 177 | 5 | p | 08707 |
| IBS133 | GATAATGGGCTTTGATGATAGG | GGTGGTGAACTAATTGCTCTCT | (ATC)8 | 60 | 156 | 3 | p | 21143 |
| IBS134 | CTTCAATCACCTGAAACTCTGA | AATATCGCTATGTTCTTGGGAC | (CT)11 | 55 | 143 | 6 | p | 00290 |
| IBS137 | TcAACAGACGTCTTCACTTACC | TCGATAGTATGATGTGAATCGC | (CTT)8 | 60 | 137 | 6 | p | 03051 |
| IBS139 | CTATGACACTTCTGAGAGGCAA | AGCCTTCTTGTTAGTTTCAAGC | (GA)7 | 60 | 196 | 9 | p | 04560 |
| IBS14 | ATCAGACATGCTTTTGTGAGAC | AGGGACTCACTTTCACTGCTAT | (TATG)7 | 60 | 175 | 4 | p | 26510 |
| IBS140 | AGCTGAGAAACGAGAGTAGGTC | CGCAAGATTACCCAACATAGTA | (ATCA)5 | 60 | 200 | 4 | p | 03513 |
| IBS141 | GAAGCAGTAGTTGTGTTGCTTT | CTCTATCTTTATCTCTTCCGGC | (CTTT)6 | 60 | 118 | 9 | p | 03406 |
| IBS144 | TCGAACGCTTTCTACACTCTT | CTGTGTTTATAGTCTCTGGCGA | (TTC)9 | 60 | 164 | 7 | p | 02819 |
| IBS145 | AAAGCCTAAAGCTCTCCCTC | ATGTTGAAAAGTCAGTGAGCAG | (TGGC)5 | 48 | 121 | 4 | p | 05627 |
| IBS146 | GCAAACCTCAAAAAGCGTAA | TAGAGGAATTGTAGGGAGTGGT | (GTCT)5 | 60 | 182 | 7 | p | 04503 |
| IBS147 | TGTGTACATGAGTTTGGTTGTG | GAAGTGCAACTAGGAAACATGA | (GCA)8 | 55 | 192 | 6 | p | 05491 |
| IBS148 | CAAGTACAGTGGCATATGGAAG | TTGCAATAGTAACCTCTGTGTG | (AATA)5 | 60 | 128 | n.d. | n.d. | 04558 |
| IBS149 | CCACCTCCTTAGGTATCAGACT | ACTACTAGCGCTGCAACCTTAT | (AGA)8 | 60 | 189 | 6 + 4 | p | 03864 |
| IBS15 | AACAACCTCAGAAACCTCAGTC | AGGTCCAGATTTGTTCTGATTC | (GTT)6-(GTT)5 |  | 151 | 5 | p | 26759 |
| IBS150 | AGTCCCTTGAATGTGTACCTCT | AGCTGCAATCATACAGTCAATC | (CT)13 | 60 | 196 | 2 + 4 | p | 09141 |
| IBS151 | ACCACCGTGAGAGGAATAAAAT | TCAGCAGTAGCTTTAACAAGGA | (TTCT)5 | 60 | 171 | 3 | p | 08069 |
| IBS152 | GCATCGGATTCATTATAGACAG | TCTCTCTAGCAACAGAAGAAACC | (GTT)8 | 60 | 100 | 6 | p | 084156_1589_1875 |
| IBS153 | GTGAAAATGTGCTCTTTAAGCC | ACACGTCTCATGTAAATGACCT | (TC)10 | 58 | 189 | 5 | p | 14758 |
| IBS154 | TGGTTGTATACCTCAACTCCAA | CTGCCTGGTATTAACACGAAA | (TTGT)5 | 60 | 196 | n.d. | n.d. | 06059/11244/FRFM4LP02S7Q30 |
| IBS156 | TTGATTCCACTATGACTTGAGC | ACACCAACCCTTATATGCTTTC | (AG)10 | 58 | 196 | 7 + 3 | p | 07833 |
| IBS158 | GAGTTGTCTGTTGTGCTTGAAT | GACTAAGCAGTGGAGAAGAAGAA | (GAT)7 | 55 | 150 | 2 | m | 11608 |
| IBS159 | GTTTGTTTCTGTGATTGGTCTG | GACACTTTTCCGTTAGACATGA | (CGAA)5 | 59 | 116 | 1 | m | 08174 |
| IBS163 | TCCACTGACAAGACTATCAAGC | GGGATTGAAGTTACGTTTTAGC | (GAAT)6 | 60 | 199 | n.d. | n.d. | 10890/21567 |
| IBS164 | CTCTTCGCCAACTCTAAATCTT | CGTACATTAAAAACAGACCGAC | (AAT)7 | 60 | 187 | 5 | p | 013594 |
| IBS166 | TCCGTCTTTCTTCTTCTTCTTC | ATACACTAACTGCATCCAAACG | (AG)10-(GAA)6 | 55 | 172 | 8 | p | 29919 |
| IBS168 | CTACACTAAAGAGCATACCCCC | TACAACTCACAACCCAGAAAAC | (TG)10 | 60 | 177 | n.d. | n.d. | 12903 |
| IBS169 | CGTACTATGTTTCCCCCATTAC | AATGCATCTACCCTCCTTACAC | (TTG)8 | 53 | 125 | 7 | p | 15078 |
| IBS170 | CAGGGCGAATAAGTCTAAAGAA | TCAACTGTGTGCATAGTTCAGT | (AG)11 | 60 | 149 | 5 | p | 16878 |
| IBS172 | CAAAAATACCCTTCCCTTGA | CTTCTAGCACCTATTTATCGGG | (AC)11 | 59 | 200 | 2 | m | 19267 |
| IBS173 | GTGAAAATGtgCTCTTTAAGCC | ACACGTCTCATGTAAATGACCT | (TC)10 | 60 | 196 | 4 | p | 14758 |
| IBS174 | AGAGAACAAAATCGGGAAGAAC | CGAAATAGAGATTGTAATGGGG | (AGA)7 | 60 | 143 | 4 | p | 23041 |
| IBS175 | GTGGAGAAGGGTAAAAGTGAGA | GCCTTTACTCCTATTCCTCATT | (TA)10 | 55 | 189 | 1 | m | 185458_1365_2719 |
| IBS18 | GCCAAGGATGAAGGATATAGAA | ACAAcCAAACTAGCTAAAAGCC | (ATG)7 | 60 | 175 | 6 | p | 00304 |
| IBS180 | AGTCAAGTCTGTCCACTCACTATT | ATCTGATGAGGAGGAAGAGAAA | (TCA)5 | 60 | 196 | n.d. | n.d. | 07665 |
| IBS181 | GAAGTTCAATAATTGGAGGAGC | CCAAGTTATATGCACAACCTGA | (TGG)5 | 60 | 180 | n.d. | n.d. | 25858 |
| IBS182 | AGAGTCATCGAACATGTCAAAC | ATCTTCGAGAGAGAGAAACCAA | (ACAT)5 | 57 | 147 | 2 | m | FRFM4LP02TCF1K |
| IBS183 | GGATTGTCAGACACAGAAGAGA | ATATGCAGCTCAGACATGAAAC | (AAC)5-(AAT)7 | 60 | 142 | n.d. | n.d. | 14078/26740 |
| IBS184 | CATTCAATTCCTTCCTTAGTCG | TTTAGTTACTGCGAAGAGACCC | (CT)6 | 60 | 113 | 1 + 13 | p | FRFM4LP02Q24VZ |
| IBS185 | CCTTGTTTATTCATCCAGCTCT | ATGTTGAAAAGTCAGTGAGCAG | (TGGC)6 | 60 | 128 | 5 | p | 27305 |
| IBS186 | CAGAAACAAGCAAAGATCTCAC | CTGTTGCTTCTCTTCTCCTTCT | (AAG)8 | 60 | 195 | 5 | p | 26702 |
| IBS187 | TTGTAGGAGTTCATCATGGCT | TTGATAGTGTTCTTCAAGGGAC | (AAAT)6 | 60 | 200 | 3 | m | 27453 |
| IBS188 | GTATTCGCTCCTAACTTTTTGG | GCTCGCCTATTTTCTCTCTCTA | (AAG)6 | 60 | 179 | 1 | m | 20602 |
| IBS189 | GACAGTTGTGGATGAAGGTTG | CCTCCTTGTTGATGAGTGAGTA | (AAAT)5 | 55 | 155 | 1 | m | FRFM4LP02QVE09 |
| IBS19 | TCCTATGAGTGCCCTAAGAATC | CTCCTTCGTCTTCTTCTTCTTC | (GGA)8 |  | 151 | 6 | p | 07544 |
| IBS194 | CAACAAAAGTCTTGGACACAAC | ACAAATCTCTATCCTTCACGCT | (ATA)7 | 58 | 166 | 1 | m | 057337_1862_2769 |
| IBS195 | TTTACTGCGTACGTTGTTTGTC | ACTCCATCACTTTTACTCCCTG | (ATT)8 | 55 | 194 | 5 | p | 065898_1800_1212 |
| IBS199 | TAACTAGGTTGCAGTGGTTTGT | ATAGGTCCATATACAATGCCAG | (ACA)7 | 60 | 155 | 10 | p | FRFM4LP02QW9DU |
| IBS20 | CATCATCaCaGCCTACATAACC | ACCTTCAGATCAACAGTTTCCT | (AAT)7-(AAC)5 | 60 | 105 | 4 | p | 12788 |
| IBS202 | CTTGAAAAAGAGGCTTCTTAGC | CTCTGCTTTCTAAACTCGGATT | (TC)9 | 60 | 148 | n.d. | n.d. | 02777 |
| IBS203 | TTGTAAGATGATGAACAGAGGC | GAGTGCAAAAGGGAGCTTATAC | (AGA)8 | 57 | 179 | 1 | m | Repeat-31127 |
| IBS204 | AGGAATTGAAGCTAAGAGCAAG | CTCTAGCAATTAAGCAAGCAAG | (AGAT)6 | 55 | 148 | 2 | p | FRFM4LP02SJKHB |
| IBS21 | ATCTTTGGGGGTTACTTCTCTT | GCTGCCAAATCACTATCAAAC | (TG)13 | 60 | 192 | n.d. | n.d. | 14216 |
| IBS22 | AATGATACCACAAGCAGAAGTG | GCTTCTTCATCTTCACTCAACTC | (TCT)8 | 60 | 200 | 5 | p | 09844 |
| IBS23 | GTTTCAACTCTCAACCAGTCAG | GGGGAAGAGAAGTTACAGAAAA | (TCT)5 | 60 | 187 | 4 | p | 14133 |
| IBS24 | AGTGCAACCATTGTAATAGCAG | TCCTTTCTTCATCATGCACTAC | (CTGC)7 | 60 | 147 | 6 | p | 13640 |
| IBS25 | AACATGATGAACACACCATCTC | GTTGCTGATGTTGAGGTAACTG | (CAG)7 | 60 | 112 | n.d. | n.d. | 15112 |
| IBS28 | ATATCTTCCAACAGTCTGCCTT | GCTTTCTGCTCTTCTTTCACTT | (TTC)8 | 60 | 176 | 3 | p | 28982 |
| IBS3 | CTTCTTTGATTGCTTCTAGCCT | ATTCATGATCTGATAGTGGTGG | (AAT)6 | 60 | 199 | 5 | p | 10385 |
| IBS30 | GGTCCTGTTAAAACAGCTCCTA | CCTGTATTTCCACAACCTACAA | (ATT)9 | 60 | 200 | 5 | p | 12752 |
| IBS33 | ATCTCTTCATACCAATCGGAAC | CAATGATAGCGGAGATTGAAG | (TCT)8 | 60 | 177 | 6 | p | 10297 |
| IBS38 | CAAATAGGAGGATACCTTAGCTG | ATAGGTTGTAGTAGGCGGAGAA | (AAAT)5 | 60 | 176 | 5 | p | 12498 |
| IBS39 | CGATGAGTAGTGAGGTGAATGT | GTCACATCTGAGAAGCATGAAC | (TTC)7 | 60 | 139 | 2 | m | 15396 |
| IBS4 | CTCTTCCTCCTCAGATTACCAC | CCAAGTTCCATATCACATCAAG | (ATC)7 | 60 | 152 | 5 | p | 13174 |
| IBS40 | AGTCTGAGCTCTAATGCTGTCA | AGCCATTGCTTGATACAAGTG | (GCT)5 | 60 | 100 | 3 | p | 23302 |
| IBS44 | TTAATACACATGCCTCTCCATC | TTCATTGTGACTGTGAGGAAG | (ATC)8 | 60 | 121 | 7 | p | 25774 |
| IBS45 | ATGAGGTTGAGGCTGAATTAGA | CACTTGAATTTTCCTTCTCTCC | (ATG)8 | 60 | 152 | 3 | p | 06772/08378 |
| IBS46 | GAGAGACAGAGATTGAAGGACC | CGGGAGTGTCAAAATCTACATA | (TC)12 | 60 | 117 | n.d. | n.d. | 08732 |
| IBS47 | GACATGTGAGCCTGTTCTTTTA | CACAGCAGGGAAGTATATGAAA | (CT)10 | 60 | 104 | 6 | p | 14914 |
| IBS48 | TGGAGTACTCGAGAGATGAGG | AAACACAGTGCTTACAGGGAAT | (CCTG)5 | 60 | 191 | 2 | p | 05831/FRFM4LP02R3R5T |
| IBS50 | CTTTAAGGTTTAATCGAGAGGG | GGACACAAAAGTCTCATTTCAC | (GA)12 | 60 | 120 | 6 | p | 00560 |
| IBS51 | ATTCTTCACACCTTCCTTCAGT | ATAAGAGGAAGAGAAAGaGGGG | (AAG)8 | 60 | 166 | 5 | p | 07425 |
| IBS52 | AGTCGGAGAGAGATATGAGGAG | TAATTTCTCGCTTGCTATGC | (GA)14 | 60 | 118 | 3 | m | FRFM4LP02QJR14 |
| IBS53 | GTTTCCACTGAAGCACAAGTT | CCACATTTTCTATTTGCCCT | (TATG)5 | 60 | 119 | 4 | p | FRFM4LP02P69D8 |
| IBS56 | AGAAGGGGGAAAAAGTCTTAAC | TCTGATCTAGGCCCAATAAACT | (AATA)5 | 60 | 193 | 3 | p | FRFM4LP02REMRA |
| IBS58 | CCAAGAGTAACGATTACTGGCT | ACAGTTGTGACCATGTGAAAGT | (TCT)9 | 60 | 198 | n.d. | n.d. | FRFM4LP02SCTT7 |
| IBS61 | GACAAACATCATCATCAGCATC | CAATCTCCTCTTTTCTACTCCC | (CAT)7 | 60 | 114 | 2 | m | FRFM4LP02RABBE |
| IBS62 | ATCGTTCACAGTGACTATCTCG | TGTCAATTGGACACCTCTGTAT | (CAGA)5 | 60 | 119 | 6 | p | FRFM4LP02SCEVS |
| IBS64 | GAGGGTTGAAGAGTTCATAAGG | AAAAGGGAGATCTTAGCTACCC | (CATA)5 | 60 | 165 | 7 | p | FRFM4LP02QG5P9 |
| IBS65 | ATGTTTTGTCAAACTCTAGGGC | CTAACATGGGTATTTTGGGA | (AAAT)6 | 60 | 198 | n.d. | n.d. | FRFM4LP02TRZN3 |
| IBS68 | CTCTCTCTCCTCCACAATTCTT | ATTTGGAGGTGAAGGTAGAGAA | (TC)11 | 60 | 162 | 2 | m | FRFM4LP02QW8PI |
| IBS71 | CTGTCTATGGCAAAATACTCCA | GGCTTGAGAGAGGTTACTGTTT | (AAAT)6 | 60 | 151 | 4 | p | FRFM4LP02QCOA0 |
| IBS72 | CTACTCTCTGCTGGTTTATCCC | CTAGTGGTCTCTCTTCCTCCAC | (AC)10 | 60 | 188 | 7 | p | FRFM4LP02PVTOZ |
| IBS73 | TTCGCTAAACTCTAGTGTCGAA | TTCTGATGTTATCCAGAGATGG | (TATT)6 | 60 | 166 | n.d. | n.d. | 17889 |
| IBS74 | GGGGAAGAATCAATCATACTCT | GAAGAAAGTGGACATGGAAGAT | (GA)11 | 60 | 176 | 3 | p | 28125 |
| IBS75 | TATGTGTGATGATGATGAGCAG | ACTTGCTTGAGCTCTTCTTTG | (AAT)8 | 60 | 153 | 3 | p | FRFM4LP02QOACU |
| IBS77 | AGCCAAGAAGGCAACAATACT | AGTTTGTGCTACCCTCGTTTAT | (AG)10 | 60 | 107 | n.d. | n.d. | FRFM4LP02RZ4TS |
| IBS78 | TGTCTCTTTCTCCTCCAATTCT | AGTGCAGAAAGGATAGGATGTT | (TCT)7 | 60 | 168 | 5 | p | FRFM4LP02RRPMA |
| IBS79 | GTTGAGATCGAATCCTTGAGTT | CCAACGATCATACTCAATACCA | (CA)11 | 60 | 153 | 5 | p | FRFM4LP02R46DJ |
| IBS80 | GGGAGGTACCACTAATCTTGAA | CCTCCTTTTCCTCTTGAATATG | (AAG)7 | 60 | 101 | 8 | p | FRFM4LP02TTTU8 |
| IBS81 | CAAAATCTCTAGCATACCCCTT | TCATCTCACTGACCAAGTATGC | (TGCA)5 | 60 | 100 | 2 | m | FRFM4LP02QG4QK |
| IBS82 | GACATAATTTGTGGGTTTAGGG | GAAATGGCAGAATGAGTAAGG | (TCA)7 | 60 | 137 | 6 | p | 27880 |
| IBS84 | CAAAGATGAAGCAAGTAAGCAG | ACTAATGTTGATCTACGGACCC | (AGAT)7 | 60 | 173 | 6 | p | FRFM4LP02TGFYQ |
| IBS85 | AACTACTCATGGGGAGAACAAC | CTAACGAAAGTTTGGACATCTG | (AC)12 | 60 | 174 | 6 | p | FRFM4LP02TK43W |
| IBS86 | AGAAACTGAAAACTAAGCTCGC | GCTATGCGTTTACAGAAACAAG | (CTT)7 | 60 | 159 | 7 | p | FRFM4LP02QL07L |
| IBS87 | CCCAAAACTACTCTTATAGCCG | AGATTCTGTATGGTTACCTGGG | (GAA)7 | 60 | 189 | 2 | p | 07237 |
| IBS88 | GATATACTGCTGCAATGGAGTG | CCAACTAAAAATAGAGGCAACC | (CT)12 | 60 | 164 | 4 | p | FRFM4LP02R8CHO |
| IBS89 | CTACGCAAAACAAAGCTATCAG | CAAATTCATCTCTTTCCCTCTC | (GA)12 | 60 | 113 | 4 | p | FRFM4LP02P13XU |
| IBS9 | ACCTAGTGCACACCATTGAGTA | GGACAACACACTTGGTTTTAAG | (TTTA)5 | 60 | 199 | 2 | m | 27731 |
| IBS90 | GTGGTGATGAGGAAACTGAAG | ACCTTCTCCAAGAACTCTTCCT | (GAT)9 | 60 | 111 | 3 | p | FRFM4LP02R04AS |
| IBS91 | GCAGCAGATGAACTATAAGCAG | CCTTCATATCACCCCTACAAGT | (ATGT)5 | 60 | 137 | 4 | p | FRFM4LP02S2H1L |
| IBS94 | TCACTTTCCTCCTTCTCTTCTC | GTTCGGCTATCTGTCAAAGAAC | (CT)11 | 60 | 134 | n.d. | n.d. | FRFM4LP02R3VJY |
| IBS95 | AGACGATGACTCCACTACCTTT | GGGCTTCTCTGCTATTATGAAC | (CCG)7 | 60 | 125 | 2 | p | FRFM4LP02SQF7O |
| IBS96 | ATGCCCAACTGTTATTACTAGG | TAGACTGTCTTTACGATGTGCC | (TTTA)5 | 60 | 104 | 2 | p | FRFM4LP02SJVPE |
| IBS97 | GTTACCAGGAATTACGAACGAT | CTCTCTACAAAAACTCACAGCG | (TG)10 | 60 | 180 | 6 | p | FRFM4LP02RPWYJ |
| IBS98 | TCTTCCTAGCTCTTGGACACAT | ATTAATATTGGTGGTGGTGGTG | (CTT)7 | 60 | 177 | 5 | p | FRFM4LP02Q26YW |
| IBS99 | GGTATGCCTCTCTTAGCTTCAT | AATCTGCAGCCATATTACTTCC | (GTAT)6 | 60 | 175 | 5 | p | FRFM4LP02RHVOT |
| IbU1 | GGCTTATGTAATAGATGCACCA | TGCTTCTATGCTCTTAAGGTTG | (TG)8 | 60 | 139 | 3 | p | 21614 |
| IbU10 | GTCTGCGTGTCCGTAGCATA | ATACGCACTTCATATACCGGC | (TC)7 | 60 | 116 | 3 | p | 23147 |
| IbU11 | AGCATGCGACTGATATTTAGG | ATATTCAAAACTGGCCGATG | (AT)7 | 60 | 112 | 5 | p | 28449 |
| IbU12 | GTGAGAGAAATCCCAAAGAGAG | ATTAGTCCTTGAAAGGCAGAGA | (TC)9 | 60 | 135 | 5 | p | 21926 |
| IbU13 | GCAACCAATCTACAGCAAACTA | CAGATAAAGTCCCCATTTCTTC | (AG)8 | 60 | 150 | 7 | p | 13836/068335_1865_2701 |
| IbU14 | GGCAAGGTTTCTCAAGTTGTTA | ACGAGATTACTTCAAATAGGGC | (TA)8 | 60 | 122 | 2 | p | 04543 |
| IbU15a | TATCAGCAAGTTCCATATGAGG | CCTTCTTCTGCTTGTGGATATT | (AT)7 | 60 | 104 | 2 | p | 04757 |
| IbU16 | CGATCTTTCTGGAAATCTGACT | CAGAGTGATCAAGAATGCAACT | (TA)7 | 60 | 178 | 2 | p | 18214 |
| IbU18 | ATTGAGTCTCTCTCTGCCTTTC | AATACTCTGACAGCGATGATTG | (TC)8 | 60 | 175 | 2 | p | 08291 |
| IbU20 | GGAGAGCAAGTGGAGAAAGTAT | ACTCCTAGACCCACAATTGAAC | (TA)7 | 60 | 177 | 6 | p | 032980_1888_2125 |
| IbU21 | CATGTACTCATTGGAAAGGATG | CACATAGATAGCAGTTTGGCAT | (AT)7 | 60 | 153 | 2 | m | 035515_1209_2230 |
| IbU22 | CACAGCGTAGAACATGTGAAG | ACAATCTAGAATTCCCACCACT | (TC)9 | 60 | 121 | 2 | p | 23102 |
| IbU23 | CTCAGACACAGAGCACTCATCT | CCTCATCACTTCCACCTAACC | (GA)13 | 60 | 115 | 7 | p | 063266_1766_2698 |
| IbU25 | CTCGGATTGATCTATGTTGCT | TGGCCATTTAGTTTCACTCA | (TA)7 | 60 | 167 | 2 | p | 075729_1350_1303 |
| IbU27 | GAAAAGCTTGTATCTTGTGTGC | CTTGGTTTCTTTCTTTCTCACC | (TC)7 | 60 | 176 | 3 | p | 106339_1135_3328 |
| IbU28 | GAAAAGTCTGGATTGCAGTTTC | GACAGATTGGATCAAAAGGGTA | (TC)10 | 60 | 138 | 3 | p | 125004_1273_0206 |
| IbU29 | AGTGCTGGCAAGTTGTTTCT | CAGTGTTCTATTGCTCTTCACTTAC | (AT)7 | 60 | 177 | 5 | p | 12980 |
| IbU30 | CTATGAAATGTTGTTAGGGCAC | ACTGTACTGGTTGGTTGGTTAT | (AG)12 | 60 | 110 | 3 | m | 150849_1843_3592 |
| IbU31 | CCGCAGAAAAAGTTCAGATT | GCAACTTTTCTTCTTCCGTAAC | (CT)12 | 60 | 158 | 6 | p | 168238_1939_0637 |
| IbU33 | TTTGAAGAAGATGAGAGCGAC | TCAGAAAGACGATACACTAGAGAGA | (TC)8 | 60 | 153 | 7 | p | 169330_1735_1048 |
| IbU34 | CTTGTGTTTGTGTTTATGTCCC | CCCCTAACGCTTCTCATATTC | (GA)11 | 60 | 148 | 4 | p | 15802 |
| IbU37 | CCTTATTTGTTACTTGTTTGATGTG | ATGCTACATGCCTAAATGTCC | (AT)9 | 60 | 110 | 5 | p | 206194_1546_1332 |
| IbU4 | GGCTGGATTCTTCATATTTAGC | GCTTAATGGATCAGTAACACGA | (GA)9 | 60 | 174 | 6 | p | 02006 |
| IbU5 | TTTCAAGGATGTGGCTAATG | CCTGCTAATATAACCCAACCTC | (AT)8 | 60 | 180 | 5 | p | 17335 |
| IbU6 | GGGGTAGAGAGAAGAGAGTGAC | CCAGGTGAGAGTGTCTTTCAA | (TC)7 | 60 | 147 | 6 | p | 00990 |
| IbU7 | GAATCTCCTTTGCTGTTTGTCT | CACATAGGCACATACTCACCTT | (AT)7-(TAGC)4 | 60 | 150 | 6 | p | 21514 |
| IbU9 | AGCAAGAAAGGTCATCATCTG | ACGAAAGAGACGAACCCTAATA | (GA)7 | 60 | 128 | 2 | p | 28704 |
